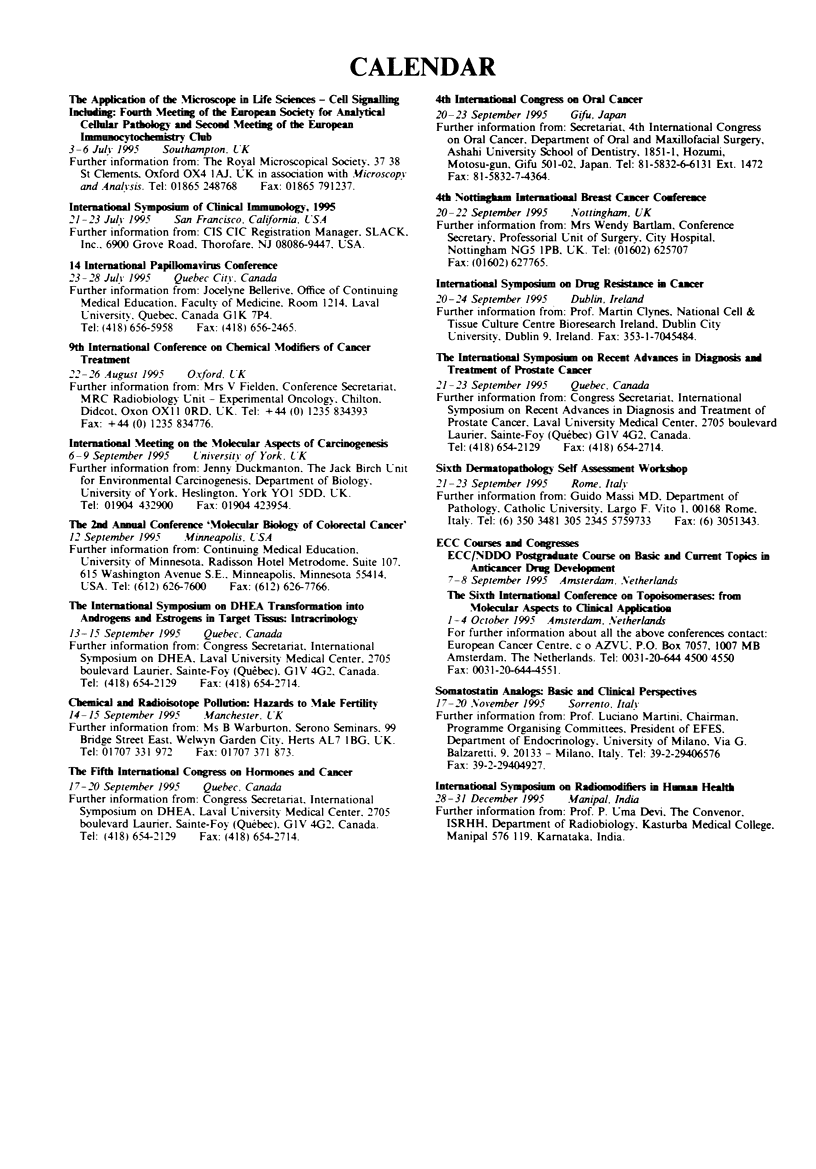# Calendar

**Published:** 1995-07

**Authors:** 


### CALENDAR

**The Application of the Microscope in Life Sciences – Cell Signalling Including: Fourth Meeting of the European Society for Analytical Cellular Pathology and Second Meeting of the European Immunocytochemistry Club**

*3–6 July 1995* *Southampton, UK*

Further information from: The Royal Microscopical Society, 37/38 St Clements, Oxford OX4 1AJ, UK in association with *Microscopy and Analysis*. Tel: 01865 248768 Fax: 01865 791237.

**International Symposium of Clinical Immunology, 1995**
*21–23 July 1995* *San Francisco, California, USA*
Further information from: CIS/CIC Registration Manager, SLACK, Inc., 6900 Grove Road, Thorofare, NJ 08086-9447, USA.

**14 International Papillomavirus Conference**
*23–28 July 1995* *Quebec City, Canada*
Further information from: Jocelyne Bellerive, Office of Continuing Medical Education, Faculty of Medicine, Room 1214, Laval University, Quebec, Canada G1K 7P4.
Tel: (418) 656-5958 Fax: (418) 656-2465.

**9th International Conference on Chemical Modifiers of Cancer Treatment**
*22–26 August 1995* *Oxford, UK*
Further information from: Mrs V Fielden, Conference Secretariat, MRC Radiobiology Unit – Experimental Oncology, Chilton, Didcot, Oxon OX11 0RD, UK. Tel: +44 (0) 1235 834393
Fax: +44 (0) 1235 834776.

**International Meeting on the Molecular Aspects of Carcinogenesis**
*6–9 September 1995* *University of York, UK*
Further information from: Jenny Duckmanton, The Jack Birch Unit for Environmental Carcinogenesis, Department of Biology, University of York, Heslington, York YO1 5DD, UK.
Tel: 01904 432900 Fax: 01904 423954.

**The 2nd Annual Conference 'Molecular Biology of Colorectal Cancer'**
*12 September 1995* *Minneapolis, USA*
Further information from: Continuing Medical Education, University of Minnesota, Radisson Hotel Metrodome, Suite 107, 615 Washington Avenue S.E., Minneapolis, Minnesota 55414, USA. Tel: (612) 626-7600 Fax: (612) 626-7766.

**The International Symposium on DHEA Transformation into Androgens and Estrogens in Target Tissus: Intracrinology**
*13–15 September 1995* *Quebec, Canada*
Further information from: Congress Secretariat, International Symposium on DHEA, Laval University Medical Center, 2705 boulevard Laurier, Sainte-Foy (Québec), G1V 4G2, Canada.
Tel: (418) 654-2129 Fax: (418) 654-2714.

**Chemical and Radioisotope Pollution: Hazards to Male Fertility**
*14–15 September 1995* *Manchester, UK*
Further information from: Ms B Warburton, Serono Seminars, 99 Bridge Street East, Welwyn Garden City, Herts AL7 1BG, UK.
Tel: 01707 331 972 Fax: 01707 371 873.

**The Fifth International Congress on Hormones and Cancer**
*17–20 September 1995* *Quebec, Canada*
Further information from: Congress Secretariat, International Symposium on DHEA, Laval University Medical Center, 2705 boulevard Laurier, Sainte-Foy (Québec), G1V 4G2, Canada.
Tel: (418) 654-2129 Fax: (418) 654-2714.

**4th International Congress on Oral Cancer**
*20–23 September 1995* *Gifu, Japan*
Further information from: Secretariat, 4th International Congress on Oral Cancer, Department of Oral and Maxillofacial Surgery, Ashahi University School of Dentistry, 1851-1, Hozumi, Motosu-gun, Gifu 501-02, Japan. Tel: 81-5832-6-6131 Ext. 1472
Fax: 81-5832-7-4364.

**4th Nottingham International Breast Cancer Conference**
*20–22 September 1995* *Nottingham, UK*
Further information from: Mrs Wendy Bartlam, Conference Secretary, Professorial Unit of Surgery, City Hospital, Nottingham NG5 1PB, UK. Tel: (01602) 625707
Fax: (01602) 627765.

**International Symposium on Drug Resistance in Cancer**
*20–24 September 1995* *Dublin, Ireland*
Further information from: Prof. Martin Clynes, National Cell & Tissue Culture Centre Bioresearch Ireland, Dublin City University, Dublin 9, Ireland. Fax: 353-1-7045484.

**The International Symposium on Recent Advances in Diagnosis and Treatment of Prostate Cancer**
*21–23 September 1995* *Quebec, Canada*
Further information from: Congress Secretariat, International Symposium on Recent Advances in Diagnosis and Treatment of Prostate Cancer, Laval University Medical Center, 2705 boulevard Laurier, Sainte-Foy (Québec) G1V 4G2, Canada.
Tel: (418) 654-2129 Fax: (418) 654-2714.

**Sixth Dermatopathology Self Assessment Workshop**

*21–23 September 1995* *Rome, Italy*

Further information from: Guido Massi MD, Department of Pathology, Catholic University, Largo F. Vito 1, 00168 Rome, Italy. Tel: (6) 350 3481/305 2345/5759733 Fax: (6) 3051343.

**ECC Courses and Congresses**

**ECC/NDDO Postgraduate Course on Basic and Current Topics in Anticancer Drug Development**

*7–8 September 1995* *Amsterdam, Netherlands*

**The Sixth International Conference on Topoisomerases: from Molecular Aspects to Clinical Application**
*1–4 October 1995* *Amsterdam, Netherlands*
For further information about all the above conferences contact: European Cancer Centre, c/o AZVU, P.O. Box 7057, 1007 MB Amsterdam, The Netherlands. Tel: 0031-20-644 4500/4550
Fax: 0031-20-644-4551.

**Somatostatin Analogs: Basic and Clinical Perspectives**
*17–20 November 1995* *Sorrento, Italy*
Further information from: Prof. Luciano Martini, Chairman, Programme Organising Committees, President of EFES, Department of Endocrinology, University of Milano, Via G. Balzaretti, 9, 20133 – Milano, Italy. Tel: 39-2-29406576
Fax: 39-2-29404927.

**International Symposium on Radiomodifiers in Human Health**
*28–31 December 1995* *Manipal, India*
Further information from: Prof. P. Uma Devi, The Convenor, ISRHH, Department of Radiobiology, Kasturba Medical College, Manipal 576 119, Karnataka, India.